# Polarization-Controlled Broad Color Palette Based on an Ultrathin One-Dimensional Resonant Grating Structure

**DOI:** 10.1038/srep40073

**Published:** 2017-01-09

**Authors:** Ishwor Koirala, Vivek Raj Shrestha, Chul-Soon Park, Sang-Shin Lee, Duk-Yong Choi

**Affiliations:** 1 Department of Electronic Engineering, Kwangwoon University, 20 Kwangwoon-ro, Nowon-Gu, Seoul 01897, South Korea; 2 School of Physics, The University of Melbourne, Melbourne, Victoria 3010, Australia; 3 Laser Physics Centre, Research School of Physics and Engineering, Australian National University, ACT 2601, Australia

## Abstract

Highly efficient polarization-tuned structural color filters, which are based on a one- dimensional resonant aluminum grating that is integrated with a silicon nitride waveguide, are proposed and demonstrated to feature a broad color palette. For such a metallic grating structure, transmissive color filtering is only feasible for the incident transverse-magnetic (TM) polarization due to its high reflection regarding the transverse-electric (TE) case; however, polarization-tuned customized colors can be efficiently achieved by optimizing the structural parameters like the duty ratio of the metallic grating. For the fabricated color filters, the transmission peaks, which are imputed to the resonance between the incident light and the guided modes that are supported by the dielectric waveguide, provided efficiencies as high as 90% and 70% for the TM and TE polarizations, respectively, as intended. Through the tailoring of the polarization, a group of filters with different grating periods were successfully exploited to produce a broad color palette spanning the entire visible band. Lastly, a nanoscale alphabetic pattern featuring a flexible combination of colorations was practically constructed via an arrangement of horizontal and vertical gratings.

Nanostructural color filters are widely regarded as a prominent alternative for the conventional dye-/pigment-based counterparts due to their conspicuous advantages in terms of compactness, spectral tailoring, environmental friendliness, compatibility with the complementary metal-oxide-semiconductor (CMOS) process, and multi-functionality^1^,^2^,^3^,^4^. For such filters, a broad color-tuning characteristic is certainly desirable for their applications in three-dimensional projection display systems, ultra-fast displays, security tags, polarization detectors, and active color pixels^5^,^6^,^7^. Devices that rely on the guided-mode resonance (GMR) effect are especially attractive for their simple design and fabrication, proper bandwidth, sound color purity, compact size, and flexible transfer characteristics^7^,^8^,^9^,^10^; however, they are mostly limited to a static case wherein the emergence of a predetermined single color is facilitated. In an attempt to obtain adaptively controlled spectral characteristics, various schemes that tapped into the adjustment of the angle of incidence or the grating period, the thermo-/electro-optic effect, and the mechanical control of the device position were previously attempted^9^,^10^,^11^,^12^,^13^; however, a high drive voltage was required in all cases, and moreover, the devices needed to be accurately aligned, thereby limiting their tuning range. Color filters that resort to the GMR in a one-dimensional (1D) grating structure were primarily studied to be operational in the transverse-magnetic (TM) mode because of their low transmission in the transverse electric (TE) mode^13^,^14^,^15^,^16^; therefore, for such GMR-based devices the generation of vivid colors for both the TM and TE modes is of prime concern in the transmissive mode, considering that polarization-sensitive coloration was only affordable in the reflective mode^17^. For a 1D metallic grating, the realization of a polarization-tuned color filter that operates in the transmissive mode is considered significantly challenging owing to its high reflection regarding the TE polarization.

In this paper, a transmissive color filter for which an ultrathin aluminum (Al)-grating structure that resorts to the GMR is exploited has been proposed and developed to provide a polarization-controlled broad color palette. The duty ratio of the metallic grating is particularly optimized to achieve enhanced resonant transmission peaks for both the TM and TE polarizations simultaneously, thereby producing vivid customized colors. The operation of the device is thoroughly analyzed in terms of the transmission response and the corresponding color properties, as well as the evolution of the resonant field profiles. A group of different color filters and an elaborate nanoscale pattern that is constructed by appropriately combining them are practically fabricated so as to confirm an arbitrary coloration set through the adjustment of the polarization and the grating period. It is predicted that the proposed devices can readily be applied to applications such as ultrafast display devices, security tags, anti-counterfeiting, and optical data storage^5^,^18^,^19^,^20^,^21^.

## Results

### Polarization-selective transmission spectra of the proposed color filter and its color response

**Figure 1:**
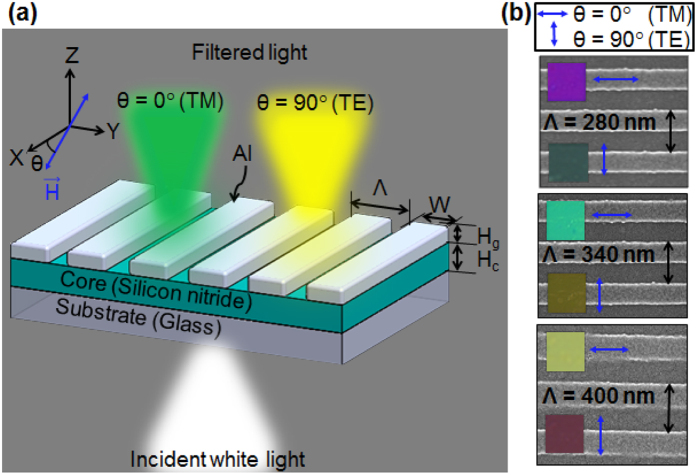
Proposed filter capitalizing on an ultrathin 1D Al resonant grating that enables a polarization-controlled broad color palette. (**a**) Schematic configuration of the proposed color filter where the incident white light is filtered into different colors depending on the polarization. (**b**) SEM images of fabricated devices with periods of Λ = 280 nm, 340 nm, and 400 nm from top to bottom. The insets display the detected vivid color images for the TM and TE polarizations, implying that the color output can be switched between purple and green, green and yellow, and yellow and magenta.

The proposed color filter incorporates a 1D Al grating of 40-nm thickness (H_g_), which is stacked on a silicon nitride core of 100-nm thickness (H_c_), as illustrated in Fig. 1(a). The chosen duty ratio of the grating, defined as the ratio of the width (W) to the period (Λ), is 0.5 to ensure that enhanced transmissions for both the TM and TE polarizations can be concurrently attained. The polarization of the incident light is indicated by the alignment of the magnetic (H) field with respect to the X-direction. The TM and TE polarizations refer to the H-field that is aligned along the X- (θ = 0°) and Y-directions (θ = 90°), respectively. The proposed device presumably exhibits transmission-type filtering characteristics that rely on the GMR effect, which transpires between the incident wave and the leaky guided modes of the waveguide that engages the silicon nitride core, as will be discussed later. For specific polarizations, the transmission of the resonant metal-dielectric structure is expressed by the following relationship^18^:


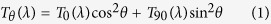


where *θ* is the polarization angle, *λ* is the free-space wavelength of light, and *T*_0_ and *T*_90_ are the transmissions for the TM and TE cases, respectively.

Figure 1(b) shows the fabricated filter tapping into an Al grating of varying periods. The insets display the microscopic vivid-color images that are associated with the devices, depending on the polarization that is marked by the blue arrow. For the devices with Λ = 280 nm, 340 nm, and 400 nm, the color output can be switched between purple and green, green and yellow, and yellow and magenta, respectively, by controlling the polarization state between the TM and the TE.

**Figure 2:**
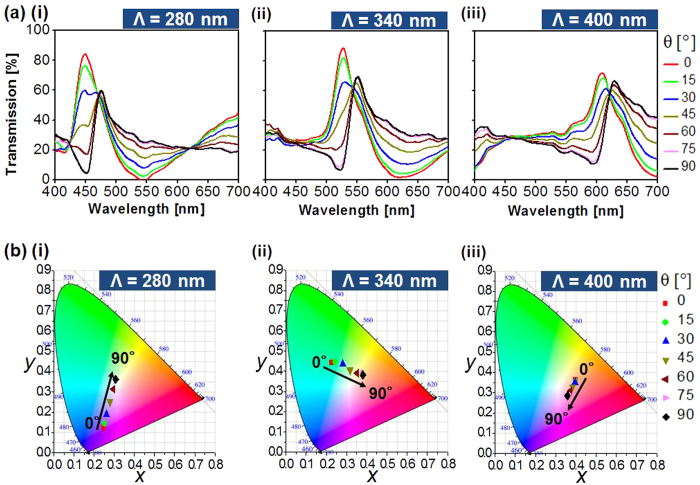
Transmission spectra of the fabricated filters and their color responses with the polarization. (**a**) Measured transmission spectra for grating periods of: (i) 280 nm, (ii) 340 nm, and (iii) 400 nm for the polarization state ranging from θ = 0° to 90°. (**b**) CIE 1931 chromaticity diagram corresponding to the spectra.

The polarization-sensitive transmission spectra are plotted in Fig. 2(a) (i) through (iii) for the cases of Λ = 280 nm, 340 nm, and 400 nm when the polarization is varied from θ = 0° to 90° in steps of 15°. The transmission spectra are checked to exhibit resonant peaks, assuming efficiencies of approximately 90% and 70% for the TM and TE polarizations, respectively. The CIE 1931 chromaticity coordinates that correspond to the polarization-dependent measured spectra, which are derived from the standard equations^7^,^22^, are shown in Fig. 2(b) (i) through (iii). The gamut of colors that is available from a pixel in relation to a particular period is represented by the line on the chromaticity diagram. The simulated transmission spectra and the corresponding color responses, which show sound correlations with the measurement results shown in Fig. 2(a,b), are displayed in Supplementary Figure S1.

### Exploration of a broad color palette resulting from polarization-mediated transmission spectra

**Figure 3:**
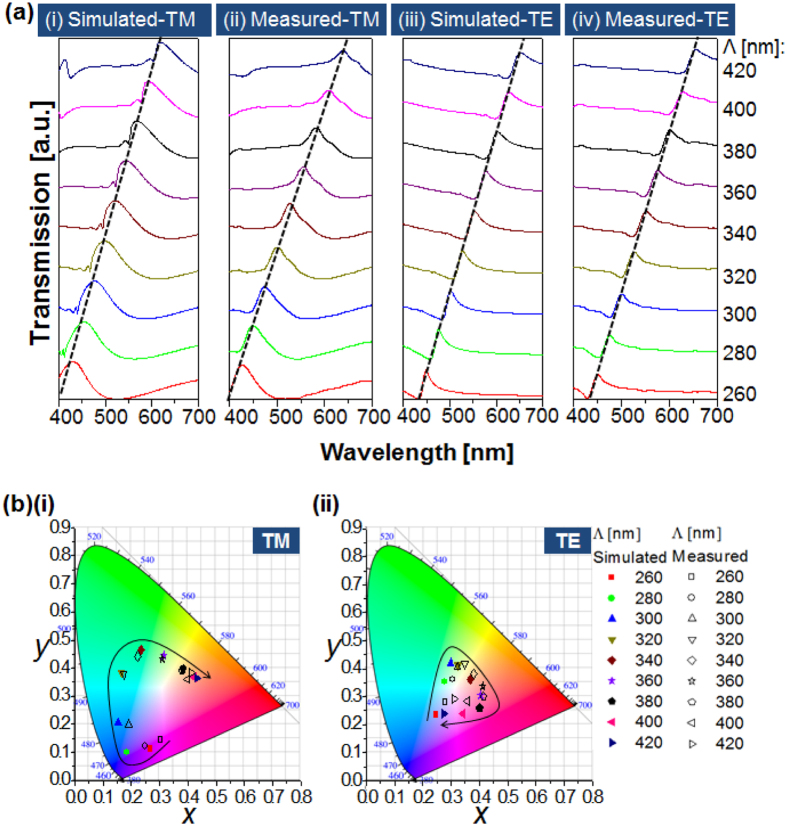
Polarization-sensitive transmission spectra and corresponding color responses with the grating period. (**a**) Simulated and measured transmission spectra for the TM and TE incident polarizations when the period is varied from 260 nm to 420 nm; here, the transmission peaks are traced by a black dashed line. (**b**) Corresponding CIE 1931 chromaticity coordinates.

**Figure 4:**
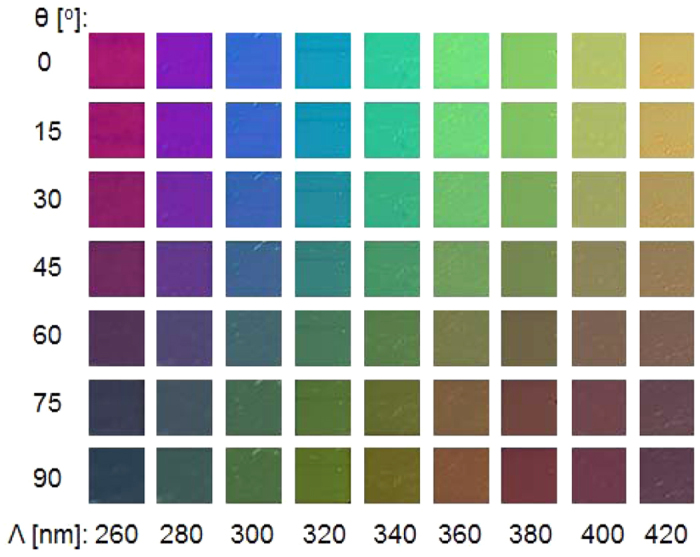
Transmission-mode bright-field microscope images with respect to the grating period and the incident polarization. For each color pixel, with dimensions of 40 μm × 40 μm, the grating period ranges from 260 nm to 420 nm, and the polarization angle is adjusted from 0° to 90°.

In an effort to accomplish a spectral performance of the device that covers the entire visible spectral band, the impact of the period of the 1D Al grating was rigorously investigated in terms of the polarization with the assistance of simulations that are based on the finite difference time domain (FDTD) method. Figure 3(a) shows the simulated and measured transmission spectra for the TM and TE polarizations when the period is scanned from 260 nm to 420 nm in increments of 20 nm. The transmission peaks are obtained simultaneously for the two polarizations, as signified by the dashed black line tracing the resonant wavelengths that run from λ = 427 nm to 639 nm for the TM and from λ = 450 nm to 655 nm for the TE when the period ranges from 260 nm to 420 nm. Figure 3(b) depicts the CIE 1931 chromaticity coordinates in response to the simulated and measured transmission spectra. It is evident from Fig. 3(b) that a set of vivid colors can be produced for both the TM and TE polarizations. However, the color purity for the TE case is relatively lower than that for the TM case. This is attributed to the fact that the transmission spectra for the TE polarization incurs a higher level of sideband compared to the TM case, thereby degrading the corresponding color purity^7^. It is noted that the sideband is effectively suppressed by the surface-plasmon resonance and neighboring Rayleigh anomaly for the TM case, unlike the TE case^16^. Each constant-period filter may act as an independent pixel that produces a polarization-mediated color palette. Figure 4 reveals the transmission mode microscope images of the manufactured filter, with dimensions of 40 μm × 40 μm, as a function of the grating period and the polarization, completing a broad color palette. The images are arranged in accordance with the period and the polarization, which are varied from 260 nm to 420 nm and from 0° to 90°, respectively.

### Mechanism that is accountable for polarization-controlled resonant transmission peaks

**Figure 5:**
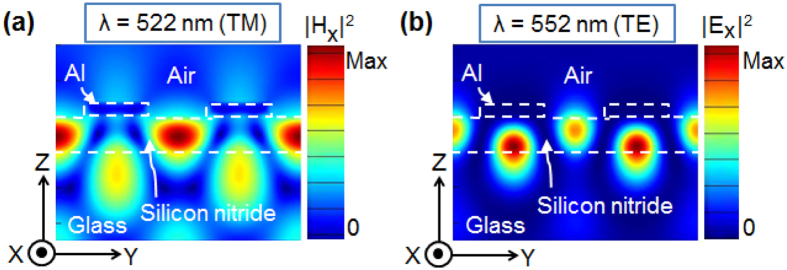
Calculated field intensity profiles in relation to the transmission peaks for the case of Λ = 340 nm. (**a**) Magnetic field intensity (|H_X_|^2^) at λ = 522 nm for the TM incidence and (**b**) electric field intensity (|E_X_|^2^) at λ = 552 nm for the TE incidence.

With the intention of elucidating the physical mechanism underlying the polarization-selective transmissions, the magnetic- and electric-field profiles were examined for the case of a typical filter with Λ = 340 nm and a duty ratio of 0.5. The H-field (|H_X_|^2^) and E-field (|E_X_|^2^) intensity profiles were monitored to be substantially reinforced in the dielectric waveguide core for the TM and TE cases, respectively, at the transmission peaks at λ = 522 nm and 552 nm, as depicted in Fig. 5(a,b). The drastically enhanced field distributions are indicative of a standing wave that develops as a result of the counter-propagating guided modes that are supported by the planar dielectric waveguide^21^.

The dependence of the spectral response and the relevant coloration of the device on the duty ratio was also elaborated, as shown in Supplementary Figures S2. The near-field profiles for the duty ratios of 0.3 and 0.7 are shown in Supplementary Figure S3. For the purpose of securing a higher transmission efficiency and an enhanced spectral shape for both the TE and TM polarizations, the duty ratio was preferentially determined as 0.5. The dependence of the transmission response was also explored in regard to the thicknesses of the Al grating and the silicon nitride core. As shown in Supplementary Figure S4, for a typical filter with a 340-nm period, the performance was substantially invariant to the thickness of the metal grating except for a slight wavelength shift, while a spectral red shift was observed with increasing thickness of the dielectric core, as shown in Supplementary Figure S5. In view of a well-defined spectral shape in conjunction with an enhanced color response, the thicknesses of the metal grating and the dielectric core were chosen as 40 nm and 100 nm, respectively.

**Figure 6:**
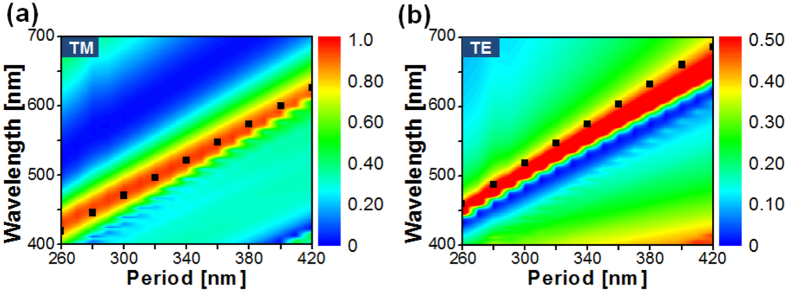
Calculated GMR conditions superimposed on the transmission spectra for different grating periods. To estimate the location of the transmission peaks, the calculated GMR conditions as indicated by the black squares are superimposed on the contour map of the simulated transmission spectra for the: (**a**) TM and (**b**) TE polarizations.

The contour map for the simulated transmission spectra is plotted in Fig. 6(a,b) for the TM and TE cases, respectively, as the grating period ranges from 260 nm to 420 nm. The calculated GMR conditions for the proposed filter are specifically superimposed on the contour map. It became evident that the GMR results can be utilized to precisely estimate the locations of the transmission peaks pertaining to the simulated spectra. The dispersion relation relating to the dielectric waveguide that is overlaid with the Al grating, which plays a role in the derivation of the GMR conditions, is given by the following equation:


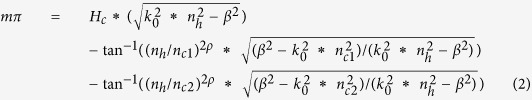


where m is the mode number, H_c_ is the thickness of the core, β is the propagation constant, n_h_ is the refractive index of the core, n_c1_ is the effective index of the upper cladding inclusive of the metallic grating and air, n_c2_ is the effective index of the substrate, and k_0_ is the wavenumber in free space. The TM and TE modes are represented by *ρ* = 1 and 0, respectively. The GMR has occurred when the phase matching is satisfied between the propagation constant β for the guided mode of the waveguide and the grating vector (G = 2π/Λ)^4^.

### Embodiment of a nanoscale pattern exhibiting a flexible combination of polarization-mediated colors

**Figure 7:**
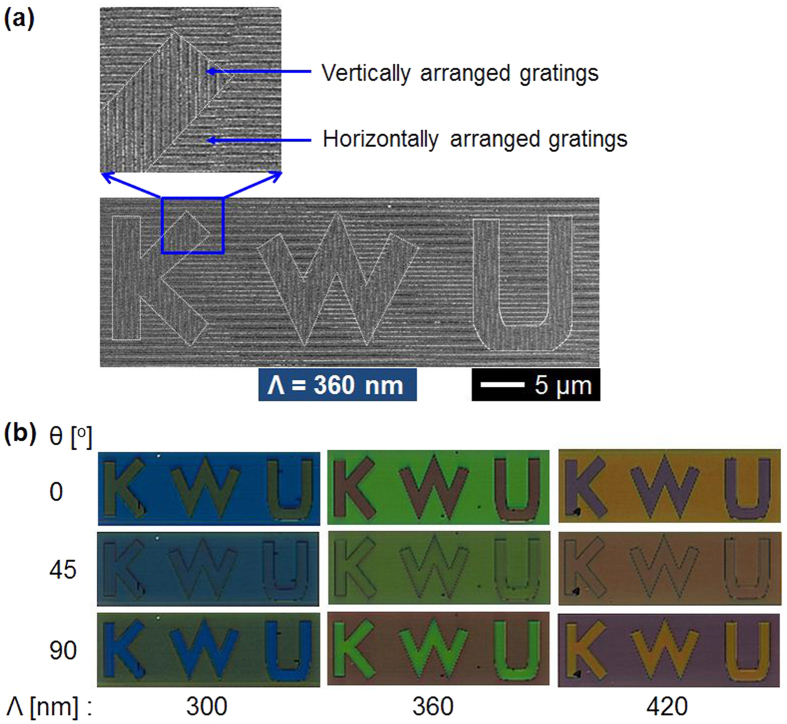
Nanoscale alphabetic pattern and its polarization-sensitive color properties. (**a**) SEM image of the nanoscale pattern of size 20 μm × 60 μm that is inscribed by a combination of horizontal and vertical Al resonant gratings with a 360-nm period. (**b**) Corresponding transmission-mode microscopic images for different periods of Λ = 300 nm, 360 nm, and 420 nm, and different polarizations of θ = 0°, 45°, and 90°.

In an effort to highlight the customized color generation of the proposed filter, a nanoscale pattern that is composed of a set of delicate alphabetic characters was concocted by inscribing vertical and horizontal resonant gratings inside and outside of rectangular boxes, respectively. The pattern was created by selecting the three grating periods of 300 nm, 360 nm, and 420 nm. Figure 7(a) depicts the SEM image of the nanoscale pattern of size 20 μm × 60 μm for the period of 360 nm, where the three letters, “K,” “W,” and “U,” as an acronym for “KwangWoon University”, are clearly seen alongside the corresponding metallic gratings. The images corresponding to the other periods are shown in Supplementary Figure S6. The microscopic color images for the pattern for different polarizations including θ = 0°, 45°, and 90° are shown in Fig. 7(b). It is noted that a flexible combination of vivid colors can be obtained by virtue of the polarization, which will be central to applications like polarization-multiplexed optical data storage.

## Discussion

Highly efficient polarization-mediated structural color filters that capitalize on an ultrathin Al grating in conjunction with a dielectric waveguide, and which provide transmission efficiencies up to 90% and 70% for the TM and TE polarizations, respectively, have been devised and evaluated. A broad color palette was realized over the entire visible band by adjusting the period of the metallic grating, as well as dynamically changing the incident polarization. To validate the ascribing of the TM and TE transmission peaks to the GMR that is initiated by the ultrathin grating in conjunction with the dielectric waveguide, both the near-field magnetic and electric intensity profiles were intensively explored together with the GMR dispersion. In particular, a nanoscale alphabetic pattern that is formed via an arrangement of horizontal and vertical gratings led to an arbitrary combination of colors, which might be a crucial feature for applications such as polarization-multiplexed optical data storage. The proposed devices will ultimately facilitate the emergence of a variety of applications encompassing security tags, display devices, image sensors, biomedical imaging techniques, and functional metasurface devices.

## Methods

### Numerical simulations

The transmission spectra and the field profiles for the color filters were investigated by means of simulations based on an FDTD tool (FDTD Solutions, Lumerical, Canada)^23^. A plane wave under a normal incidence was introduced by taking into account the refractive indices of silicon nitride, Al, and SiO_2_^24^. A unit cell satisfying the periodic boundary conditions was taken advantage to mimic the periodically arranged Al nanowires.

### Device fabrication

The proposed color filters were designed and manufactured to exhibit dimensions of 40 μm × 40 μm. A 100-nm thick silicon nitride film was deposited on a glass substrate using plasma enhanced chemical vapor deposition (PECVD) (Oxford, Plasmalab System 100). A 40-nm thick Al film was then deposited with the help of an electron-beam evaporator (Temescal BJD-2000 E-beam/Thermal Evaporator System), which was subsequently patterned through an electron-beam lithography system (RAITH 150) for which a positive resist of ZEP520A was adopted, and dry etching was then performed in a plasma etcher (Versaline LL ICP Etching System) under a gas mixture of Cl_2_, BCl_3_, and Ar.

### Optical characterization

The completed Al pattern was visually evaluated under a high-resolution field emission scanning electron microscope (FESEM S-4800, Hitachi). The transmission spectra were checked for different polarizations through the launching of a halogen-lamp (HL-2000-FHSA, Ocean Optics) collimated beam, which was properly polarized through a calcite crystal polarizer (GTH 10M-A, Thorlabs), toward the prepared filter that had been mounted on a motorized rotation stage via a focusing lens. The optical output was captured by a spectrometer (Avaspec-3648, Avantes) via a multimode fiber. The images that are related to each pixel of the color filter were captured using a digital microscope (Leica DM6000 M).

## Additional Information

**How to cite this article**: Koirala, I. *et al*. Polarization-Controlled Broad Color Palette Based on an Ultrathin One-Dimensional Resonant Grating Structure. *Sci. Rep.* **7**, 40073; doi: 10.1038/srep40073 (2017).

**Publisher's note:** Springer Nature remains neutral with regard to jurisdictional claims in published maps and institutional affiliations.

## Supplementary Material

Supplementary Information
