# Supplementary material for: Polarization-Controlled Broad Color Palette Based on an Ultrathin One-Dimensional Resonant Grating Structure
Source: Sci Rep. 2017 Jan 9;7:40073. doi: 10.1038/srep40073 (PMC5220310; doi:10.1038/srep40073)
Supplement: Supplementary Information [file srep40073-s1.pdf]

SUPPLEMENTARY INFORMATION

Polarization-Controlled Broad Color Palette Based on an Ultrathin One-Dimensional Resonant Grating Structure

Ishwor Koirala[1], Vivek Raj Shrestha[1,2], Chul-Soon Park[1], Sang-Shin Lee[1,] and Duk-Yong Choi[3]*

[1]Department of Electronic Engineering, Kwangwoon University, 20 Kwangwoon-ro, Nowon-gu, Seoul 01897, South Korea

[2]School of Physics, The University of Melbourne, Melbourne, Victoria 3010, Australia

[3]Laser Physics Centre, Research School of Physics and Engineering, Australian National University, Canberra ACT 2601, Australia

[*]E-mail: slee@kw.ac.kr

Keywords: Nanoscale devices, Displays, Optical Physics

1. Simulated transmission spectra and corresponding color response for the polarization-sensitive structural filter with respective to grating period.

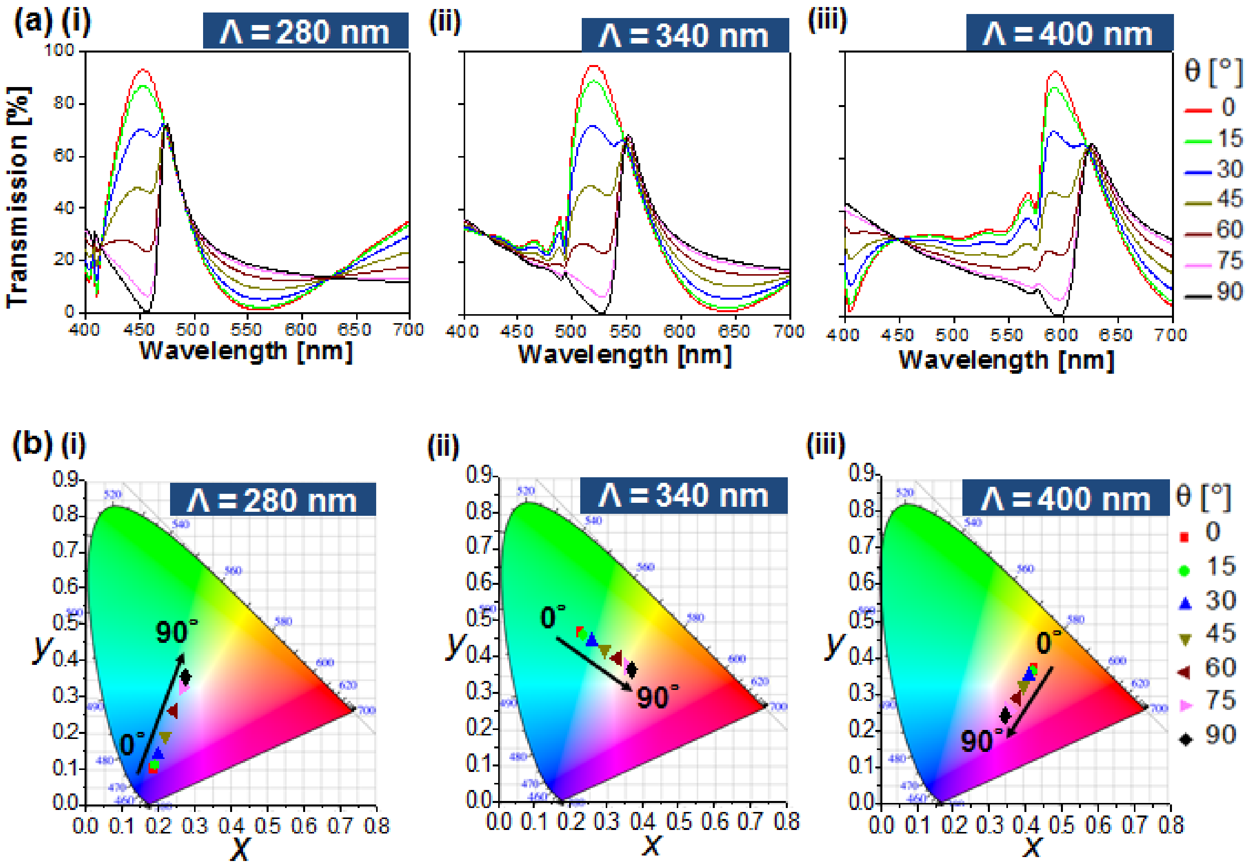

Supplementary Figure S1. (**a**) Simulated transmission spectra of the filters with grating periods of: (i) 280 nm, (ii) 340 nm, and (iii) 400 nm for polarization angles ranging from θ = 0° to 90°. (**b**) Corresponding CIE 1931 chromaticity diagram.

2. Device performance in terms of the duty ratio.

The influence of the duty ratio on the transmission efficiency and coloration for both the TM and TE polarizations is shown in Supplementary Figure S2. The devices for a duty ratio of 0.5 are discovered to exhibit a nearly optimum performance in terms of the transmission efficiency and the sideband for both the TM and TE polarizations, which are basically related to the color tuning, and this is compared with the cases for the duty ratios of 0.3 and 0.7.

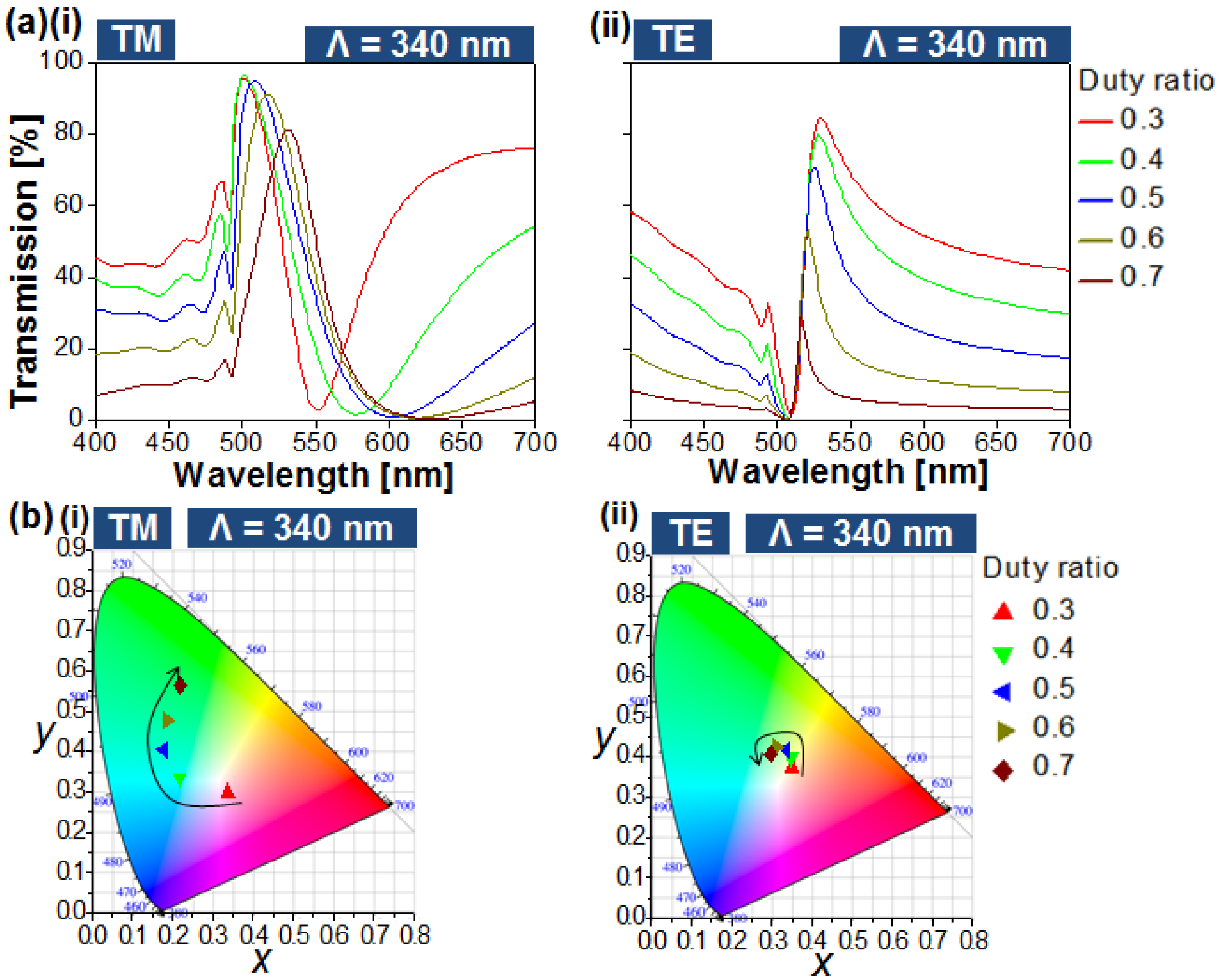

Supplementary Figure S2. (**a**) Simulated transmission spectra for the grating period of 340 nm for the TM and TE polarizations with the duty ratio ranging from 0.3 to 0.7. (**b**) Corresponding CIE 1931 chromaticity diagram.

3. Field profiles in terms of the duty ratio.

For the devices with duty ratios of 0.3 and 0.7, the GMR occurrences at the resonant wavelengths, which is supported by the field intensity distribution, are shown in Figures S3(**a**) and S3(**b**) for the TM and TE polarizations, respectively.

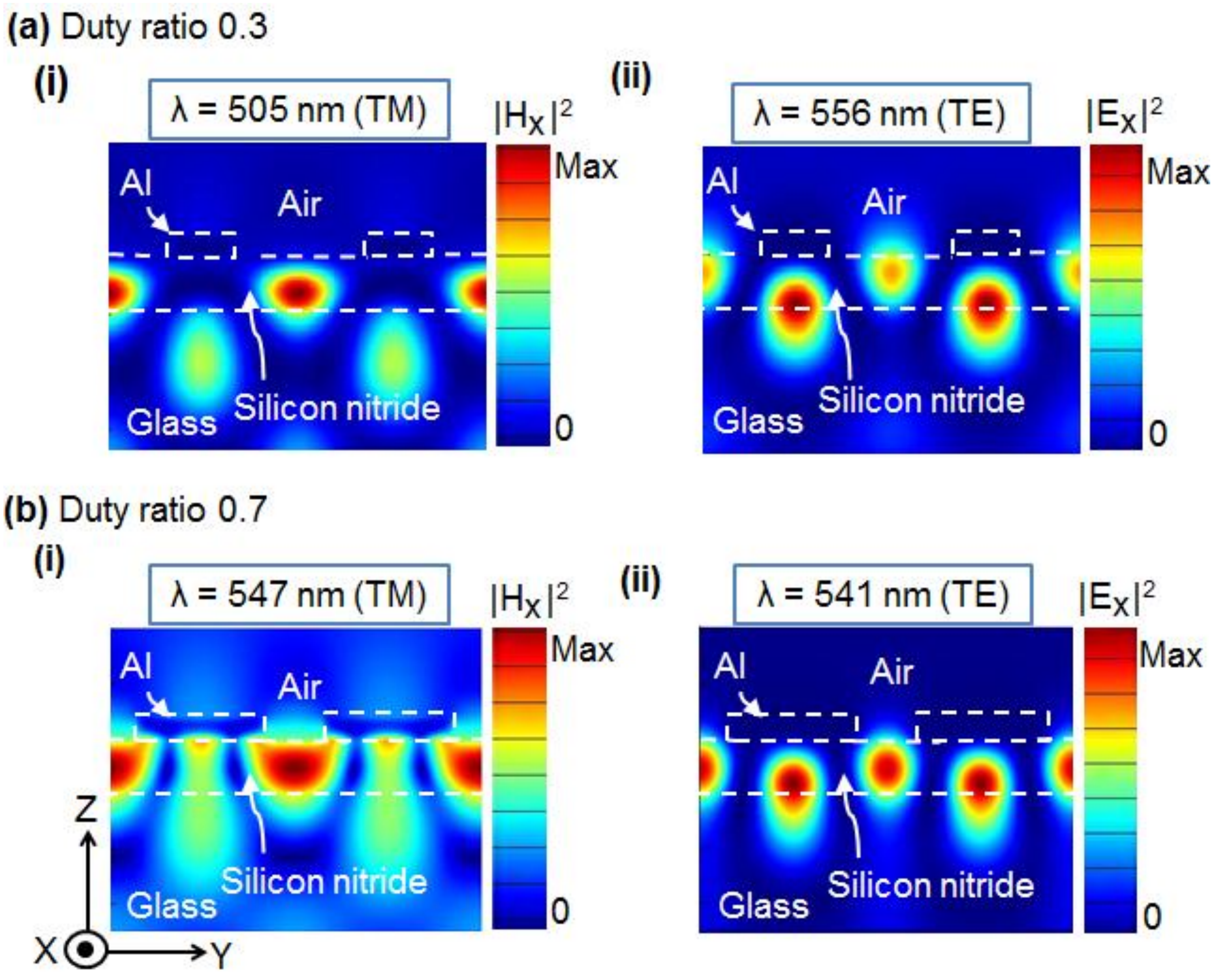

Supplementary Figure S3. Simulated resonant field intensity distributions for the filter with Λ = 340 nm for duty ratios of (**a**) **0.3** and (**b**) **0.7:** (**a**)(i) Magnetic-field intensity ($|H_X|^2$), (**a**)(ii) electric-field intensity ($|E_X|^2$), (**b**)(i) magnetic-field intensity ($|H_X|^2$), and (**b**)(ii) electric-field intensity ($|E_X|^2$)) for the TM and TE polarizations, respectively.

4. Effect of the thickness of the Al grating on the transmission spectra and the corresponding color response.

The effect of the thickness of the Al grating on the spectra and the corresponding chromaticity coordinates is addressed, as shown in Figure S4. It is confirmed the performance of the proposed device exhibits a relaxed structural tolerance in terms of the grating thickness.

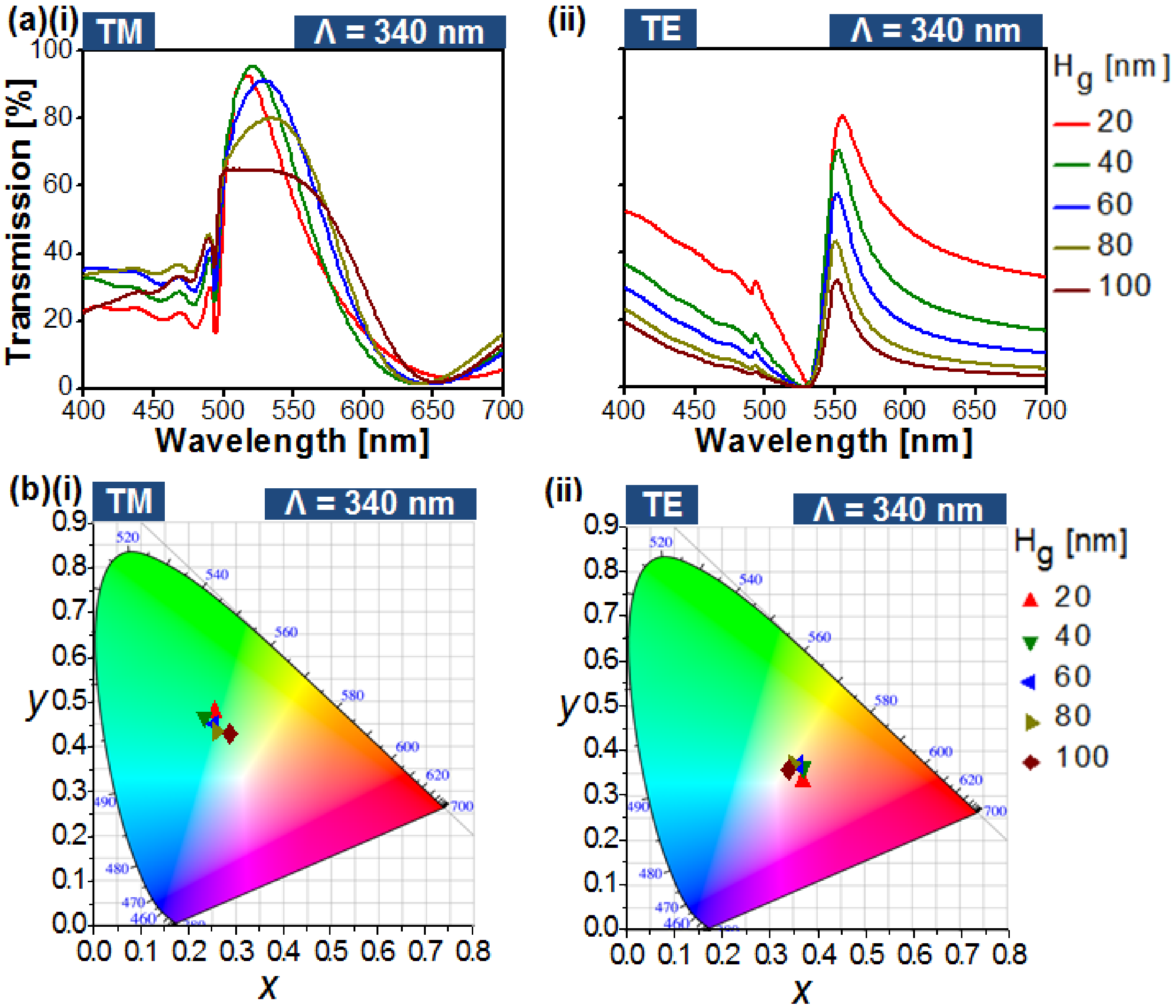

Supplementary Figure S4. Effect of the thickness of the Al grating on the transmission spectra and the color response. (**a**) Simulated spectra for the grating period of 340 nm, when the Al thickness ranges from 20 to 100 nm for the TM and TE polarizations, and (**b**) the corresponding CIE 1931 chromaticity diagrams.

5. Effect of the thickness of the silicon nitride core on the transmission spectra and the color response.

The effect of the thickness of the silicon nitride core on the spectra and the color response is investigated as shown in Figure S5. The spectra are observed to red shift with increasing thickness of the dielectric core. In light of a well-defined spectral shape and enhanced performance, the thickness has been set at 100 nm.

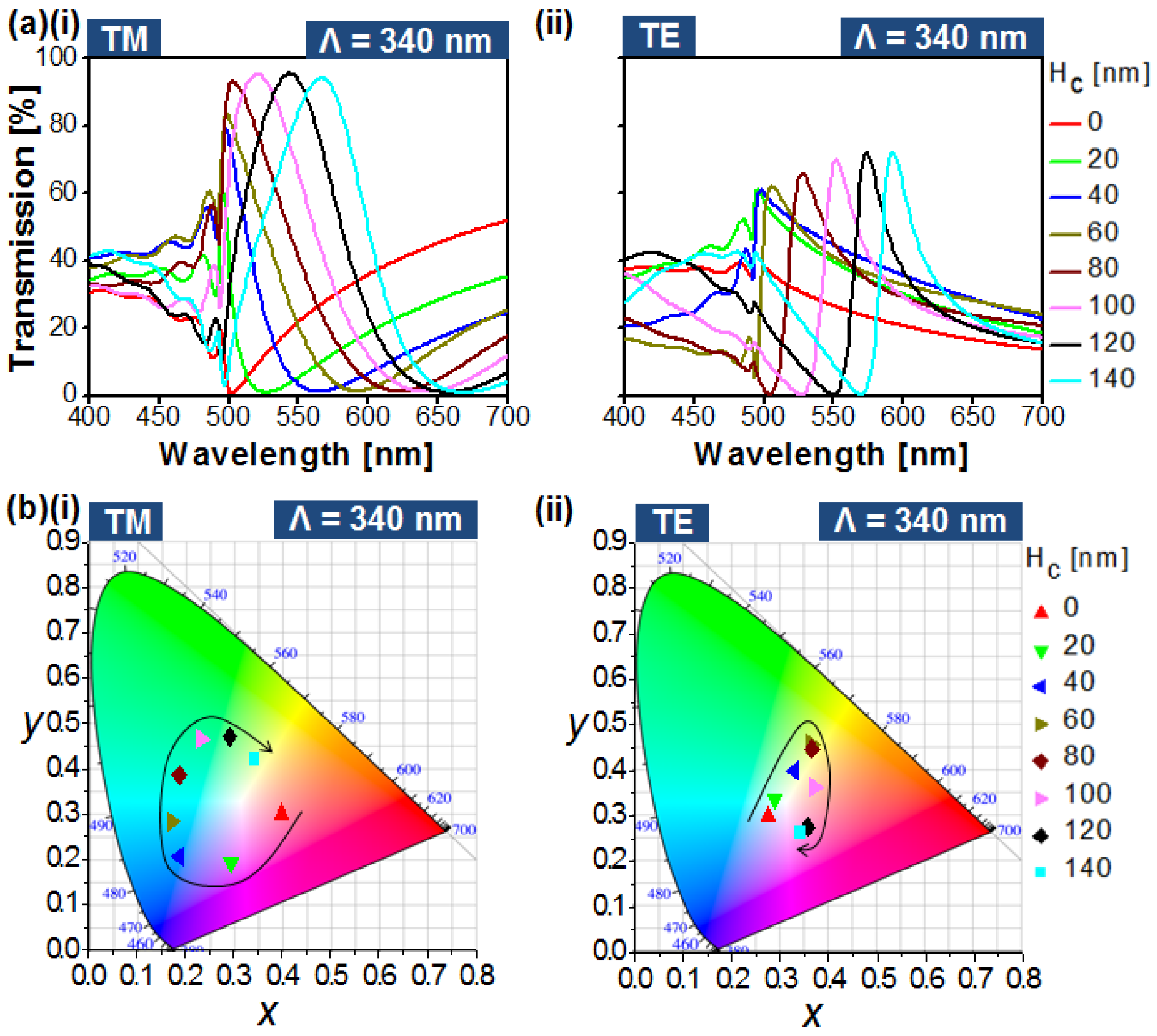

Supplementary Figure S5. Effect of the thickness of the silicon nitride core on the transmission spectra and the color response. (**a**) Simulated spectra for the grating period of 340 nm, when the dielectric thickness is varied from 0 to 140 nm for the TM and TE polarizations, and (**b**) the corresponding CIE 1931 chromaticity diagrams.

6. SEM images of the constructed alphabetic characters.

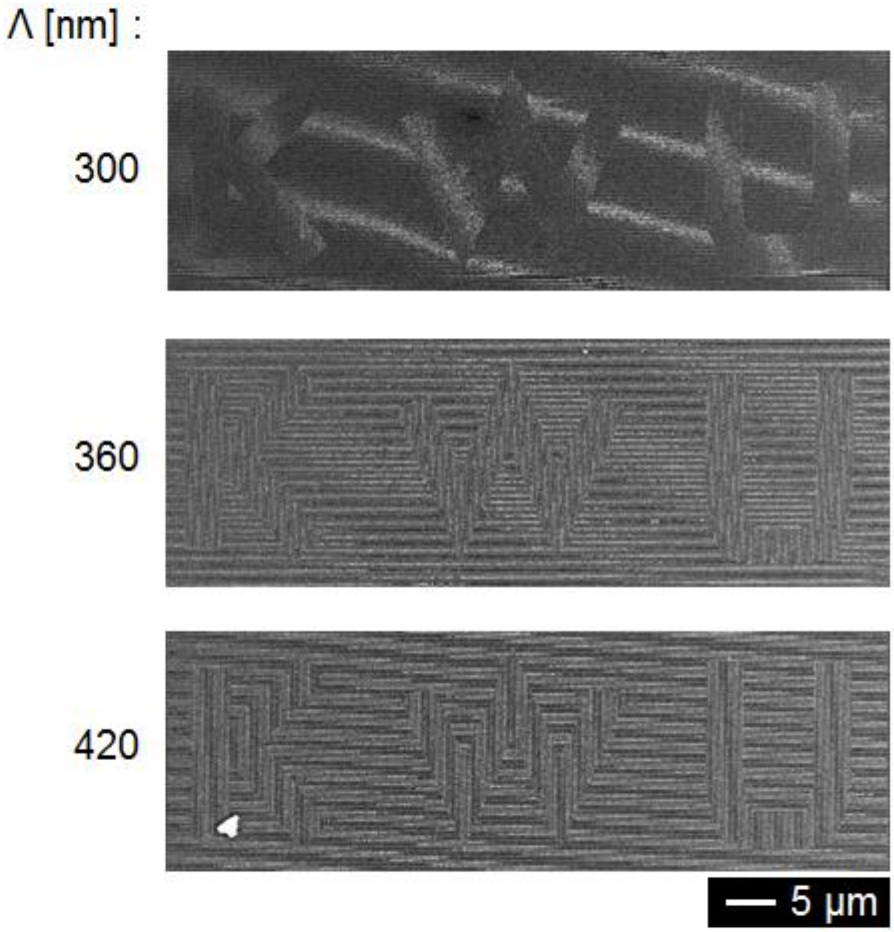

Supplementary Figure S6. SEM images of the nanoscale alphabetic characters of “K,” “W,” and “U,” with dimensions of 20 µm × 60 µm, which consist of a combination of vertical and horizontal gratings for the characters and the background, respectively, for the cases of Λ = 300 nm, 360 nm, and 420 nm.
